# The Critical Role of Autophagy and Phagocytosis in the Aging Brain

**DOI:** 10.3390/ijms26010057

**Published:** 2024-12-25

**Authors:** Stephen C. Bondy, Meixia Wu

**Affiliations:** 1 Department of Occupational and Environmental Health and Department of Medicine, University of California, Irvine, CA 92697, USA; 2 Evergreen World ADHC, Westminster, CA 92844, USA; meixiawu1997@yahoo.com

**Keywords:** brain aging, phagocytosis, autophagy, mitophagy, neurodegeneration

## Abstract

As the organism ages, there is a decline in effective energy supply, and this retards the ability to elaborate new proteins. The consequences of this are especially marked in the gradual decline in brain function. The senescence of cells and their constituent organelles is ultimately the cause of aging of the entire nervous system. What is less immediately obvious is that brain aging is also accompanied by the failure of catabolic events that lead to the removal of non-functional cells and ineffective subcellular components. The removal of non-working cellular and subcellular elements within the brain is essential in order to allow the appearance of fresh cells and organelles with a full range of capacities. Thus, the maintenance of operative mechanisms for the dispersal of failed tissue components is important, and its diminished capacity with aging is a significant contributory factor to the onset and progression of age-related neurological disorder. This report discusses the mechanisms underlying autophagy and phagocytosis and how these can be adversely modulated as aging proceeds. The means by which the effective recycling of cellular components may be reinstated in the aged brain are considered.

## 1. Introduction

The management of the disposition of poorly or non-functioning cells and subcellular components in nervous tissue is achieved primarily by two major disposal systems. One of these, phagocytosis, involves the incursion of glial cells performing phagocytic ingestion and removal of materials. These phagocytic cells are mostly microglia, but astroglia can also carry out this function. Whole cells, or parts of cells such as synapses, can be removed in this manner. Such phagocytosis plays a role in the sculpting of functioning neuronal circuitry, but if excessive and less discriminate, can enhance the pathological changes associated with both normal brain aging and neurodegenerative disease [1]. Extruded material from cells such as myelin fragments can be eliminated by this means, and inadequate phagocytic removal of myelin debris has been associated with multiple sclerosis [2]. Phagocytic activity is also a major means of destroying invasive pathogens.

The other system of clearance is by cells undertaking digestion of their own malfunctioning constituents, by autophagy. An important subcomponent of this is mitophagy whereby damaged mitochondria can be removed, allowing replacement by fresh ones by mitogenesis. Brain aging is associated with declining mitochondrial functioning, resulting in failure to supply the energy required for anabolic events [3]. Thus, one mechanism involves the recognition of damage via external monitoring by phagocytic cells, while the other requires intracellular self-monitoring. In either case, the overall outcome can lead to improved organ operation. In the absence of clearance of such tissue debris by phagocytic and autophagic means, the new biosynthesis of cells and organelles is inhibited [4]. The processes of breakdown of depleted cellular constituents and their replacement by fresh elements are closely linked, and brain aging is associated with failure of this linkage. There is evidently a linkage between the synthesis of fresh mitochondria and the destruction of their damaged or aged variants. Several of the dietary and exercise strategies that are reported to accelerate mitogenesis have also been found to lead to enhanced mitophagy. Impaired mitophagy during aging appears to stimulate mitogenesis. The mediating signal may be the reactive oxygen species produced by defective mitochondria [5].

In the case of the nervous system, the development of neurodegenerative diseases is associated with even further malfunction of this regenerative cycle [6]. Removal of non-functioning intracellular materials can also lead to improved resistance to disease. Studies with *Drosophila* reveal that inhibition of autophagy prevents effective control of Zika virus infection of the brain and increases the mortality of infected insects, while activation of autophagy by rapamycin is protective [7]. Rapamycin acts to inhibit the mammalian target of rapamycin (mTOR), and such suppression can lengthen the lifespan of a wide range of species including vertebrates, nematodes, and single-celled organisms. Inhibition of mTOR signaling not only stimulates autophagy but retards microglial activation and furthers polarization of microglia toward the anti-inflammatory M2 phenotype. This prevention of microglial transition to the M1 form inhibits inflammation [8]. Many micronutrients derived from natural sources also inhibit mTOR, suggesting that this may be their major mode of action in promoting health [9].

There are indications that effective mitophagy is an important component of the immune response and can protect against infection. For example, mitophagy can reduce the severity of life-threatening inflammation incurred during sepsis. Intracellular pathogens and inflammatory pathways can often be counteracted by stimulation of mitophagy during severe infection [10]. Immune mediators can promote or inhabit autophagy, while immune signaling trajectories can be regulated by autophagy. Thus, there is a bidirectional regulatory relationship between immune signaling trajectories and autophagy. One generalization is that cytokine pathways that induce autophagy are often inhibited by autophagy. This negative feedback loop prevents excessive activation of inflammation and helps to maintain a degree of homeostasis [11].

## 2. Glial Phagocytosis

### 2.1. Protective Attributes of Microglial Phagocytosis

There is growing evidence the aging brain accumulates cell types that have diminished effectiveness in carrying out their functions. Defective cells within nervous tissue have to be removed before they can be replaced by fresh cells with improved operating capacity.

The process of removing dysfunctional or dead apoptic cells is largely brought about by microglia after their transformation into an active phagocytic state. In addition to acting in a phagocytic manner by removing invasive microbes and viruses, such microglia can remove intrinsic cells that are damaged. This phagocytotic activity is also a key means of pruning superfluous synapses and dendrites of neurons during development, allowing the emergence of operational interactive pathways. Reduction in the strength of input from cell bodies increases the likelihood of their terminals being removed by phagocytosis [12]. Exosomes secreted by neurons can further promote the capacity of microglia to remove degenerating neurites [13]. This allows for the sculpting and maintenance of essential neural pathways. Entire neurons that are supernumerary can be removed during such a sculpting process [1]. Cell debris and amyloid aggregates and damaged myelin fragments can similarly be removed [14]. Even the inflammatory M1 form of microglia has also been reported to be capable of these activities [15].

The AIM2 (melanoma2) inflammasome senses damage-associated changes and then advances microglial conversion to the M1 form, enabling inflammation and apoptosis. Genetic knockout of this factor leads to improved spatial memory and increased dendritic branching in mice [16]. Overexpression of AIM2 leads to both increased microglial inflammatory and phagocytic activity. This phagocytosis is abnormal and leads to synaptic engulfment, thereby reducing the functional capacity of neurons. This reveals how inflammation and phagocytosis can act either together or in opposition. An increased activity of AIM2 is associated with the aging process [17].

Defective microglial phagocytosis has been associated with behavioral abnormality. Mice deficient in transmembrane protein 59 (TMEM59), a protein regulating microglial functioning, have a diminished ability to selectively engulf redundant synapses and an increased content of dendritic spines. Such dysregulated pruning reflects a deficit in successful phagocytosis and leads to elevated levels of excitatory synaptic activity, which can adversely affect behavioral capacity [18]. Activation of the beta-cell receptor CD22 inhibits phagocytosis by microglia and is upregulated with age. Impeding this receptor in aged mice with an antibody enhances the clearance of myelin debris and of amyloid-β peptide aggregates, and also improves behavioral functioning [19].

There is good evidence from gene deletion studies that microglia are essential for the maintenance of normal brain function and a significant component of this is likely to be due to the phagocytic properties of microglia. Dysfunction of autophagy is associated with the pathogenesis of Alzheimer’s disease (AD), and the depression of autophagy disrupts cognitive function [20]. In animal models of Alzheimer’s disease, where a specific gene is deleted, leading to the complete absence of microglia, this brings about cerebral amyloid angiopathy and shortened longevity. These changes can be blocked by the injection of a suspension of microglia derived from wild-type animals [18]. However, depression of phagocytosis by removal of microglia has also been reported to lead to reduced engulfment of synapses, resulting in neuroprotection and enhanced cognition [21] (Gabande-Rodriguez). The molecular events causing these changes are complex and only partially understood, and this may account for several apparently conflicting findings.

Mitophagy and phagocytosis are linked in that damaged mitochondria may be extruded from neurons and taken up for phagocytic digestion by adjacent glia [22].

### 2.2. Harmful Effects of Microglial Phagocytosis

As aging progresses, the efficiency of the phagocytic process gradually declines, and prolonged overactivation of microglial cells by continual disposition of an increasing load of poorly digestible myelin and misfolded protein fragments can lead to cytotoxicity. This leads to lipofuscin deposition within microglia and their excessive production of inflammatory mediators, which furthers senescence-associated impaired brain functioning [23]. The cognitive decline found in Alzheimer’s disease is at least in part attributable to the increased microglial phagocytosis of synapses [24]. Microglia appear able to clear extracellular Aβ in AD, but aging has been associated with inappropriate rather than merely diminished rate of phagocytosis. Such aberrant phagocytosis can result in the destruction of healthy synapses and memory loss [25]. This misplaced action has been proposed to play a role in normal brain aging and more so in neurodegenerative diseases [1], where the phagocytosis of healthy synapses may further the advancement of Alzheimer’s disease (AD) [26]. Changes in microglial responses that are encountered in several neurodegenerative diseases may adversely affect the balance between the beneficial and harmful consequences of phagocytosis [27]. The inflammatory cascade initiated by glial NF-kB seems instrumental in bringing about the conversion of beneficial phagocytic events to harmful neuroinflammation [12]. This illustrates the need to consider the qualitative aspects of phagocytosis as well as its intensity. Overall, these apparently conflicting reports suggest that a key balance point has to be reached to allow optimization of the aging process, by enabling a moderate but not excessive degree of phagocytosis. Knockout of genes related to phagocytosis retards neurodegenerative processes in animal models of AD, and so the causal relationship of such apparently simultaneous events is not readily determined. In fact, inhibition of phagocytosis has been suggested as a means of slowing the progression of AD [26]. The intensity of the phagocytic process seems to be a determinant of its utility and follows a biphasic shape.

These findings lead to the question of how microglial phagocytosis fails with aging. Microglia actively phagocytosing Ab deposits in a mouse AD model, ultimately secreting a range of inflammatory cytokines [28]. It may be that the attempted digestion of an overabundance of aggregated Ab peptides leads microglia toward a chronic inflammatory state. Such a connection between failed phagocytosis and excessive inflammation may define the onset of a deviant form of microglial response. Senescent cells which appear during normal aging also secrete inflammatory cytokines and are able to activate inflammasomes. Their phagocytic destruction will retard the overall aging process [29]. The aberrant regulation of autophagy found with senescence may involve lysosomal failure and accumulation of misfolded protein complexes, and this is a contributing factor of neurodegenerative events [30]. In summary, the quality and quantity of phagocytosis are both determinants of the value of this process in furthering events that support or prevent positive aging.

### 2.3. Astrocytes

The continual removal of redundant synapses throughout life is largely performed by astrocytes [31]. APOE2, a protective APOE allele against AD, amplifies the phagocytic proficiency of astrocytes. In aged APO-2 knock-in animals, the number of senescent synapses in the hippocampus is reduced [31,32]. Synaptic plasticity is especially critical in the hippocampus, as it is involved in the formation of new memories.

Astrocytes may play an important role in the phagocytosis of amyloid plaques, both by modulation of microglial activity and by direct phagocytotic action. However, it is unclear whether such activity can lead to astrocytic death [31]. It has been proposed that the astrocytic role is more focused on maintaining effective synaptic activity, while microglia are more oriented toward actually regulating the formation or removal of synapses, thus directly impacting behavioral function [33].

## 3. Autophagy Within the Brain

### 3.1. Beneficial Aspects of Autophagy

The removal of damaged proteins and organelles within the cell is an important source of self-regulation during senescence [34]. As mature neurons can survive a long time without the ability to divide, autophagy is especially important in the brain. There are several distinct systems of autophagy. Macroautophagy occurs by way of autophagosomal engulfment and degradation of ineffective subcellular materials. After autophagy, the resulting phagosomes fuse with proteolytic lysosomes, which can break down protein debris and thus allow recycling of amino acid constituents to the cell [34]. The ubiquitin–proteasome (UPS) system is more selective as it is concerned with the removal of those proteins that have been tagged for degradation by ubiquitinoylation. Chaperone-mediated autophagy (CMA) is also specific in that it involves guidance by chaperone proteins of materials destined for destruction into proteolytic lysosomes [35].

Although autophagy consists of replacement of inadequate intracellular components, it can also lead directly to cell death [36]. Apoptosis effects cell death by disassembly of the cell in a stepwise manner. While this process primarily utilizes caspases to break down intracellular proteins, the two mechanisms for solving the problem of what to do with cells that exhibit reduced usefulness make use of many of the same pathways. Autophagy is generally initially inhibitory of apoptosis and may represent an attempt to salvage a cell, which if unsuccessful may ultimately trigger a switch to apoptosis [37].

Broken-down constituents are removed to prevent them from becoming deleterious permanent intracellular inclusions, and their constituents are available for anabolic recycling and elaboration of new, functional materials. In the brain, this can mean the prevention or clearance of deposited protein complexes associated with neurodegenerative disease, such as amyloid peptide and tau depositions associated with Alzheimer’s disease (AD), and parkin, found in Parkinson’s disease. Autophagy is an important cellular response to various stress stimuli and can be categorized into less selective and more selective autophagy. Recent studies have indicated that both types of autophagy are involved in AD pathology [38].

Several neurodegenerative diseases are characterized by malfunctioning of autophagy, and this is reflected by the lower presence of specific markers for autophagy (AGT5 protein) and mitophagy (parkin) in the plasma from patients with several types of dementia or mild cognitive impairment, reflecting a significant down-regulation of autophagy and mitophagy pathways in these groups of patients [39]. Human genetic variants, with mutations impeding the pathways leading to autophagy, exhibit a wide range of specific and serious neurological disorders. These include learning difficulties, ataxia, tremor, hearing loss, optic atrophy, congenital encephalopathy, ataxia, encephalopathy, seizures, and cerebellar hypoplasia [40].

Mitophagy, the destruction of ineffective aged mitochondria, is an important component of autophagy. Mitochondrial quality declines with age and especially so in age-related neurological disease such as AD [38,41]. The selective removal of these is essential to allow replication of unimpaired mitochondria by fission. This elimination is reduced with age and more severely compromised in the disease state. Enhanced mitophagy can depress AD symptoms [42].

Autophagy is diminished in AD brains, and restoring the extent of autophagy in animal models of AD can be neuroprotective [43]. Mitochondrial fusion and fission also play a part in maintaining mitochondrial health. Fission is essential for mitochondrial biogenesis, but excessive levels of fission can result in fragmented and ineffective mitochondria. Fusion allows an exchange of mitochondrial, while fusion causes a mixing of constituents between mitochondria. This exchange of mitochondrial ingredients can lead to restoration of mitochondrial function [38]. In Alzheimer’s disease, the presence of indigestible proteinaceous materials, such as Aβ and phosphorylated tau, leads to excess oxidant and inflammatory activity, which can result in the inhibition of mitophagy [44].

A limitation on the effectiveness of autophagy with the advancement of aging is that the expression of autophagy-related genes declines with age. Since the overexpression of these genes has been found to increase the lifespan of *C. elegans* [45], this decline may be one of the primary initiators of aging. Overall, autophagy is often positive and its induction in *C. elegans* extends its lifespan [46]. The autophagic responses of CD4+ T-cells from individuals belonging to families with unusual longevity have a greater induced autophagic activity relative to an age-matched control group. Such active T-cells appear to result in increased resistance to infection and lessened autoimmunity, and this may relate to the greater lifespan of such families [47]. Caloric restriction, which is known to extend longevity, has been found to augment autophagy. This amplification may be mediated by the ability of caloric restriction to inhibit mTOR function [48].

While promotion of autophagy is frequently found useful in the treatment of animal models of PD and AD, chronic stress can impair hippocampal neurogenesis by way of induction of a detrimental form of autophagy [49]. A more beneficial form of hippocampal autophagy and mitophagy can be fostered by various pharmacological agents as well as lifestyle factors.

### 3.2. Transition to Adverse Forms of Autophagy

In addition to qualitative differences between various autophagic events, the magnitude and temporal scale of autophagy are also important variables in determining outcomes. For example, while autophagy can initially effectively contribute to the clearance of aggregated proteins in the early stages of a neurodegenerative disease, as the disease state develops, the effectiveness of protease degradation decreases and leads to an increased level of autophagy, which then results in cell death [50,51].

The original concept that autophagy serves to subdue senescence by the removal of non-functional intracellular materials is obviously an incomplete perspective, and a much more complex situation is gradually unfolding. Rather than neurosenescence leading to a simple diminution of autophagy, brain aging is accompanied by the appearance of a defective form of autophagy which may be less selective. Diseases that involve premature aging, like ataxia telangiectasia, xeroderma pigmentosum, and Cockayne syndrome, all have reduced levels of mitophagy but an increase in overall non-specific autophagy [30]. In parallel to this, it may be that the opposing effects of autophagy, which can either inhibit or stimulate senescence-associated secretory phenotype (SASP) formation, are due a focused form of autophagy targeting and inhibiting SASP, while a more random form of autophagy leads to further SASP production [52]. Other adverse effects of autophagy have been found in various experimental systems. Extended restraint stress of rats inhibits hippocampal neurogenesis, and this was associated with the autophagic death of neural stem cells [49].

Ineffective neuronal autophagy has been linked to depression, bipolar disorder, and schizophrenia, leading to the suggestion that increasing the intensity of autophagy might be beneficial and concurrently lead to a range of improvements in overall systemic health. This highlights the distinct needs of cerebral tissue with a low rate of mitosis. However, enhancing autophagosome formation, although effective in disease animal models, may have risk in conditions when autophagosome clearance is defective [53].

Deviant autophagy can accelerate aging by stimulating the synthesis of senescence-associated secretory proteins [52]. Autophagy dysfunction is characteristic of most neurodegenerative diseases related to aging where indigestible proteinaceous inclusions are present [54]. There may be a bidirectional pathological interaction between the insoluble forms of tau protein found in AD and aberrant autophagy, and this is accompanied by chronic inflammation. Continuing neuroinflammation leading to the chronic presence of inflammatory cytokines may initiate these changes toward aberrant or unsuccessful autophagy [38].

Aberrant autophagy is not merely a quantitative lessening of activity but has qualitative aspects. It is often associated with the failure of autophagosome maturation. This leads to autophagosomes being unable to fuse with lysosomes and consequently accumulating. This abnormality is found in many neurodegenerative diseases [55]. Such deviant autophagosomes, whose progression is stalled, are larger than usual and represent another undesirable intracellular inclusion [56]. In mouse models of Alzheimer’s disease, such blockage in autophagosome maturation was observed and was associated with diminished cognitive functioning [20]. Nevertheless, the overall turnover of mitochondria diminishes with age [57] as does the appearance of new, functioning autophagosomes [58]. The question has been raised as to the whether there is only a minimal decline in autophagy during healthy aging. The opposite is certainly true in that defective autophagy reduces longevity and can result in progeria [34].

While there is good evidence for the utility of stimulating autophagy, some laboratories have found that chronic microglial depletion can increase the complexity of neural networks and their connectivity in both normal and AD-modeling mouse brains. Unexpectedly, rather than impairing memory-related tasks, microglial depletion was actually improved by reduced synaptic sculpting [59]. To sum up, microglial elimination has been shown to have both neuroprotective and detrimental effects and distinguishing between these is not yet well understood [60]. These conflicting findings may reflect the tendency of useful autophagy to be converted into a defective form of autophagy, especially with age and neurodegenerative conditions. They may also reflect the many adverse roles of microglia in establishing a state of sustained inflammation with aging [61]. Although microglia undergo replication, they remain vulnerable to senescence with declining function [62]. Microglial inflammatory action is magnified and extended in the aged brain, leading to behavioral deficits and chronic, unfocused, and harmful inflammation [63]. The continuous presence of microglia in the M1 configuration results in chronic but unserviceable inflammation [61]. This situation is even more pronounced and irreversible in AD and several other neurodegenerative disorders [64]. Microglia are very varied and exist in many diverse forms. The role of microglia is further complicated because they have many different functions and exist in more than merely activated M1 and quiescent M2 states. Overall, the M2 state tends to be more phagocytic than the M1 configuration, but the phagocytic state is neither an inflammatory nor a resting state and so does not fall into this simplified classification.

### 3.3. Systems Regulating Autophagy and Mitophagy

mi-RNAs are important regulators of autophagy. Inhibition of microRNAs such as miR-34a, which suppress autophagic activity, can slow the progression of disease in an animal model of AD, by allowing more autophagy. miR-34 content is decreased in long-lived dietary-restricted mice [65]. Other microRNAs such as miR-331-3p and miR-9-5p, when overexpressed, led to reduced autophagy and resulted in elevated amyloid deposition in a mouse model of AD [66]. Physical activity induced by swimming or wheel-running can also inhibit aging in both natural aging and experimentally induced aging, in part by way of modulation of mi-RNAs [67]. There are clear pro-longevity effects of exercise for cognitive, cerebrovascular, and systemic health, and these also involve enhanced mitophagy in experimental animals [68]. Exercise-induced activation of the autophagy-enhancing miR-130a leads to increased autophagy [69]. Urolithin is a metabolite formed in the gut from ellagic acid, a component found in various fruits such as pomegranates, raspberries, and walnuts. In a D-galactose model of brain aging, administration of urolithin leads to increased autophagy, thereby retarding the development of galactose-induced aging [70]. Urolithins have been found to extend the lifespan of animal models and have anti-inflammatory effects in humans [71]. A metabolic trajectory initiated by urolithin occurs by way of upregulation of miR-34a expression. This leads to the activation of Silencing information regulator 2 related enzyme (SIRT1), resulting in the inhibition of mTOR activation. These events are permissive of increased autophagy [66]. AMP-activated protein kinase (AMPK), another major promoter of autophagy, is increased by agents such as icariin, a plant-derived flavonoid thought to reduce the rate of neurosenescence by improving brain function and enhancing neuronal autophagy. This effect is also mediated by blocking mTOR activation [72].

TREM2, a receptor expressed on microglia, is a major regulator of microglial function. Lacking this receptor causes diminished phagocytosis and excessive proliferation of dendrites and synapses in young animals, which leads to abnormal behavior [73]. In the case of humans bearing a TREM2 defective loss-of-function variant, there is an increased risk of developing AD. Antibody-effected TREM2 activation is currently being considered as a therapeutic means of treating this type of AD [73]. The TREM2 complex on the surface of microglia mediates between recognizing a variety of extracellular agents reflecting impaired well-being, and the initiation of intracellular signaling cascades. Materials such as damage-associated molecular pattern molecules (DAMPs), cell debris, and Aβ peptides are detected, and the resulting signal trajectories trigger several beneficial reactions including promotion of autophagy and phagocytosis [74].

Another neuroprotective regulator is mitochondrial PTEN-induced kinase 1 (PINK1). Mitochondria that are defective are removed by mitophagy after their depolarized state is detected by PTEN-induced PINK1 [75]. This enzyme then initiates selective mitophagy of defective mitochondria. Overexpression of PINK1 promotes a more general form of autophagy that can extend to the dispersal of Tau protein accumulations found in AD. Enhancing PINK1 content has therefore been proposed as having therapeutic potential in the treatment of AD [76].

## 4. Environmental and Therapeutic Measures to Enhance Mitophagy and Autophagy

Several means have been proposed to further the rate of breakdown and clearance or re-assimilation of damaged cells and intracellular organelles. Modification of factors relating to the patterns of living are a primary and most inexpensive and readily achievable means of retarding the speed of senescence. They include stress management, caloric moderation, and adoption of a consistent sleep regimen and exercise [77]. All of these modifications enhance effective autophagy. There is considerable evidence that mitophagy levels decline with age, and thus, preventing this decline may reduce the speed of neurodegeneration and extend the healthy lifespan. It is noteworthy that autophagy levels do not decline with age in the naked mole rat, a species which has an unusually long life expectancy [78]. The expansion of pharmacological tools to maintain the intensity of mitophagy with age may be useful. A range of phytochemicals and zoochemicals are described as promoting mitophagy. Examples of this class of compound include resveratrol [79], curcumin, spermidine and taurine [80], catechins [81], and melatonin [82]. Taurine supplementation acts in an anti-inflammatory manner [80]. Many of these phytochemicals are found in Mediterranean and Okinawan diets. In epidemiological studies, these diets have been related to extended longevity and a decreasing risk for age-related disease relative to the diet typical of Western countries [83]. A man-made pharmaceutical, metformin, used in the treatment of diabetes, also has this broad set of attributes, and has protective properties in animal models of neurodegeneration [84,85].

Such pharmacological agents and phytochemicals have differing sites of action in promoting mitophagy. Metformin and spermidine act by way of inducing PINK1, which effects Parkin translocation to depolarized mitochondria and induces mitophagy. Rapamycin enhances mitophagy by blocking mTOR, resveratrol effects are mediated by stimulation of SIRT1, which activates the PINK1/Parkin pathway and many relevant transcription factors, while urolithin induces the formation of a range of mitophagy-associated proteins. Curcumin depolarizes defective mitochondria, which can lead to their detection by mitophagic mediators, or the apoptosis of the entire cell [86,87]. Removal of mitochondria that are no longer functioning leads to enhanced mitogenesis, thus enabling their replacement by fresh fully operational mitochondria [79]. Members of this list commonly have anti-inflammatory and antioxidant properties and are reported to delay senescence in the brain. This complicates understanding of the mechanisms by which they can also promote autophagy. Determining the chain of causality between these closely linked events is elusive. It may be that these attributes are in fact, temporally inseparable. It has been proposed that the antioxidant properties of these molecules might relate to their mitophagy- and mitogenesis-stimulating properties in the nervous system [83]. Clear separation of autophagy from anti-inflammatory and antioxidant events cannot readily be accomplished since they are often found together. However, some agents acting primarily as antioxidants such as pantothenate, a-tocopherol, and α-lipoate, which may have fewer secondary qualities, cannot promote autophagy, indicating that antioxidant and autophagic pathways are not identical [88]. That consequences of stimulation of mitophagy are invariably beneficial may represent an oversimplification as there are also aberrant forms of mitophagy [89].

The question as to whether classical antioxidants interact with autophagy in a positive or negative manner remains unclear. Reactive oxygen species can inhibit pathways such as mTOR and activate the AMPK signaling cascade, thereby enhancing autophagy. However, lower levels of reactive oxygen species can inhibit autophagy. Antioxidants may well have a bidirectional effect on autophagy depending on their concentration [90,91]. Many thiol antioxidants such as glutathione and cystamine can impair autophagy, and the potential utility of some antioxidants in the treatment of neurodegenerative diseases may be negated. In fact, such compounds can elevate the content of the proteinaceous aggregates characterizing several neurodegenerative disorders [92]. In light of this, the indiscriminate use of antioxidants as a means of ameliorating neurodegenerative decline may need reconsideration.

Recent evidence indicates that the digestive system and the nervous system are mutually interactive in a crucial manner [93]. A high-fat diet not only results in obesity and a tendency toward diabetes but also depresses autophagy within the brain and impairs learning and memory processes. This is in part mediated by activation of mTOR. Consequential pathological changes are apparent in the hippocampus [94]. This is likely to be part of the trajectory by which an inappropriate diet can impact brain function. Conversely, significant protective effects of dietary constituents on brain function can also be systemically mediated. For example, dietary administration of dimethylene blue or resveratrol alters the profile of the gut microbiome, and this change can cause activation of the cerebral Nrf2/ARE signaling pathway within the brain. This then promotes the induction of genes leading to antioxidant and anti-inflammatory factors, and also promotes factors stimulating mitophagy, thus bringing about the synthesis of new mitochondria in the aged mouse brain. The behavioral consequences of this are improved long- and short-term memory [79].

## 5. Conclusions

The appropriate removal of flawed cell organelles or whole cells from the aging brain can be beneficial in retarding the rate of progression of neurodegenerative changes. Such clearance provides materials and space for their replacement by new operational constituents. This biogenesis process can occur even in advanced age. However, there are several limitations that narrow the constructive nature of these processes. One of these is quantitative in that excessive levels of apoptosis, pruning, or organelle digestion can obviously become detrimental. The intensity of autophagy is an important factor in determining whether a beneficial or harmful outcome will result. Thus, a moderate elevation in autophagy increases the longevity of flies, while a greater enhancement of autophagy diminishes life expectancy [95].

**Figure 1:**
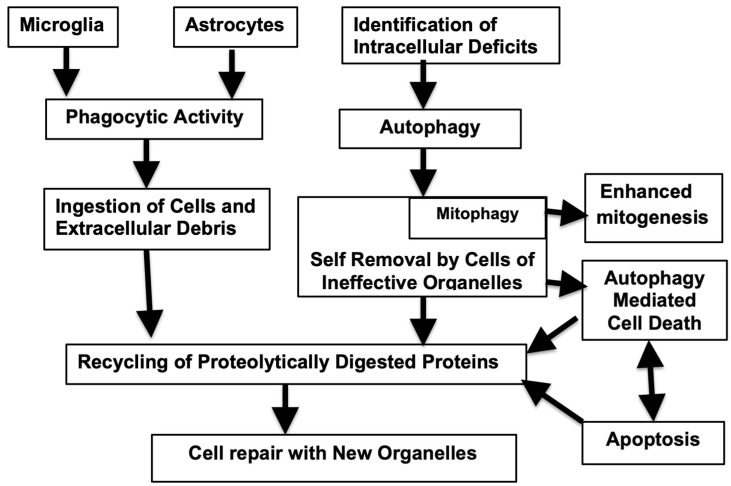
Autophagy and phagocytosis: breakdown of failed cells and cellular constituents resulting in beneficial metabolic changes and maintenance of cellular efficiency.

**Figure 2:**
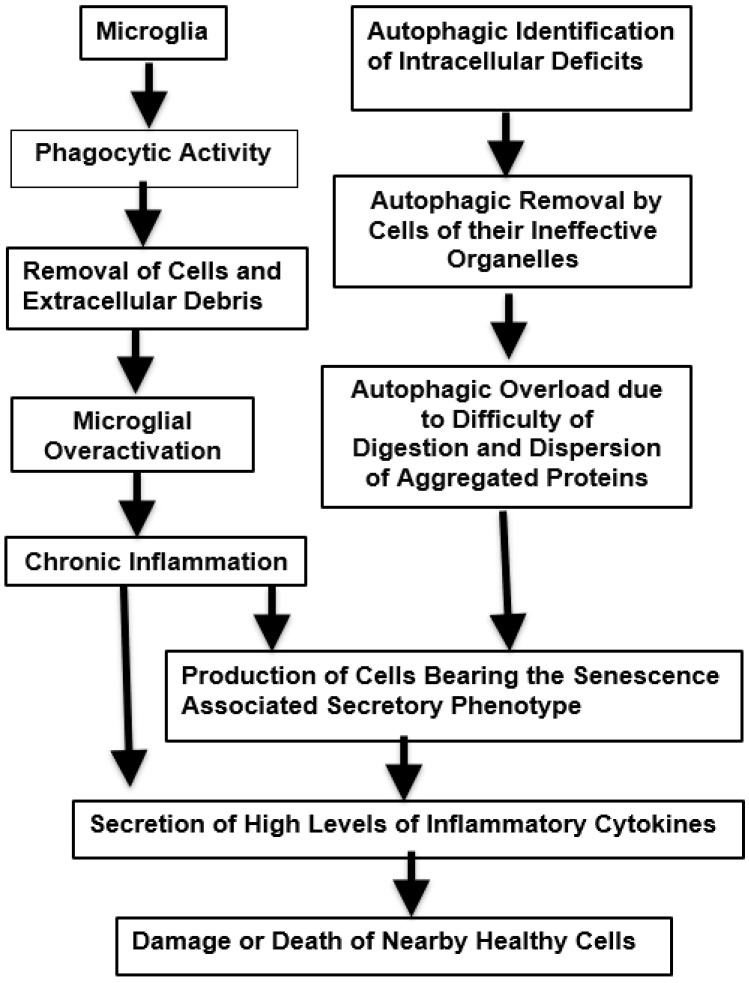
Phagocytic and autophagic pathways that may experience excessive demand, leading to inflammation and potentially harmful neurodegenerative changes.

Another serious shortcoming of the removal of cells or their constituents is qualitative. In this case, the nature of these processes can become abnormal. This may take place when the phagocytic response is overwhelmed by the presence of inclusions within the cells that gradually accumulate and are difficult to disperse. Many pathological misfolded proteinaceous aggregates found in neurodegenerative diseases are not present in the a-helical configuration but form a network due to the cross-linking of peptide chains to form a web. This b-sheet structure is not readily cleared by proteases. In response, the stressed cells bearing this lesion phagocytes may change over to an SASP-synthesizing variant. These senescence-associated proteins can then activate microglia to the inflammatory M1 state and the generation of inflammasomes, changes that can accelerate the aging process [29]. Thus, in AD, high levels of autophagy are present together with a reduction in lysosomal proteolytic activity of insoluble complexes [96]. When these cells finally die, the aggregated proteins become extracellular and further trigger futile microglial activity and more neuroinflammation. A simplified version of events associated with effective phagocytic and autophagic responses are illustrated in Figure 1, while the age-related pathological changes in these are shown in Figure 2.

Despite these limitations and concerns over the potential dual role of autophagy [97], there are no reports of the application of therapeutic pharmacological and phytochemical materials, leading to the production of excessive and harmful levels of autophagy and phagocytosis, or causing a transition to detrimental variants of these processes during aging. Thus, developing strategies to enhance these processes seems a safe means by which to attempt impedance of neurodegenerative progression and promotion of healthy longevity [98].

**Figure 3:**
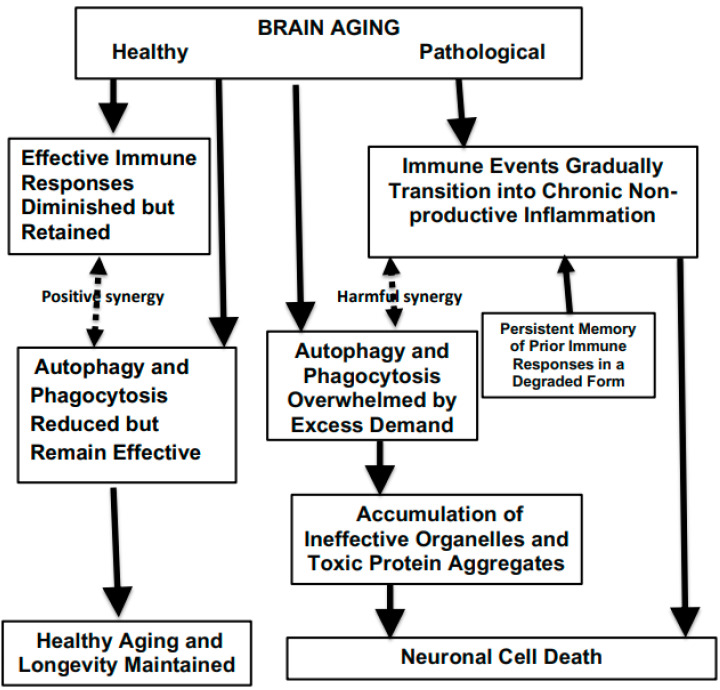
Critical decision points distinguishing healthy brain aging from pathological age-related developments.

Neuropathological aging takes place when targeted phagocytic, autophagic, and inflammatory events gradually develop an increasingly growing diffuse penumbra, which is unfocused and devoid of utility. The bifurcation between normal and pathological neurodegeneration is summarized in Figure 3. This figure also illustrates the interactions between the failure to remove damaged tissues and immune malfunction.

## Data Availability

No new data were created or analyzed in this study.

## References

[1] Vilalta A., Brown G.C. (2018). Neurophagy, the phagocytosis of live neurons and synapses by glia, contributes to brain development and disease. FEBS J..

[2] Sen M.K., Mahns D.A., Coorssen J.R., Shortland P.J. (2022). The roles of microglia and astrocytes in phagocytosis and myelination: Insights from the cuprizone model of multiple sclerosis. Glia.

[3] Bondy S.C. (2024). Mitochondrial Dysfunction as the Major Basis of Brain Aging. Biomolecules.

[4] Anding A.L., Baehrecke E.H. (2017). Cleaning House: Selective Autophagy of Organelles. Dev. Cell..

[5] Diniz L.P., Araujo A.P.B., Carvalho C.F., Matias I., de Sá Hayashide L., Marques M., Pessoa B., Andrade C.B.V., Vargas G., Queiroz D.D. (2024). Accumulation of damaged mitochondria in aging astrocytes due to mitophagy dysfunction: Implications for susceptibility to mitochondrial stress. Biochim. Biophys. Acta Mol. Basis Dis..

[6] Mizushima N., Komatsu M. (2011). Autophagy: Renovation of cells and tissues. Cell.

[7] Liu Y., Cherry S. (2019). Zika virus infection activates sting-dependent antiviral autophagy in the Drosophila brain. Autophagy.

[8] Chen Y., Chen J., Xing Z., Peng C., Li D. (2024). Autophagy in Neuroinflammation: A Focus on Epigenetic Regulation. Aging Dis..

[9] Wu Q., Gao Z.J., Yu X., Wang P. (2022). Dietary regulation in health and disease. Signal Transduct. Target. Ther..

[10] Ma L., Han T., Zhan Y.A. (2024). Mechanism and role of mitophagy in the development of severe infection. Cell Death Discov..

[11] Cadwell K. (2016). Crosstalk between autophagy and inflammatory signalling pathways: Balancing defence and homeostasis. Nat. Rev. Immunol..

[12] Sochocka M., Diniz B.S., Leszek J. (2017). Inflamm. Response CNS: Friend or Foe?. Mol. Neurobiol..

[13] Bahrini I., Song J.H., Diez D., Hanayama R. (2015). Neuronal exosomes facilitate synaptic pruning by up-regulating complement factors in microglia. Sci. Rep..

[14] Shobin E., Bowley M.P., Estrada L.I., Heyworth N.C., Orczykowski M.E., Eldridge S.A., Calderazzo S.M., Mortazavi F., Moore T.L., Rosene D.L. (2017). Microglia activation and phagocytosis: Relationship with aging and cognitive impairment in the rhesus monkey. Geroscience..

[15] Sierra A., Abiega O., Shahraz A., Neumann H. (2013). Janus-faced microglia: Beneficial and detrimental consequences of microglial phagocytosis. Front. Cell Neurosci..

[16] Chen J., Shu S., Chen Y., Liu Z., Yu L., Yang L., Xu Y., Zhang M. (2019). AIM2 deletion promotes neuroplasticity and spatial memory of mice. Brain Res. Bull..

[17] Ye L., Shu S., Jia J., Sun M., Xu S., Bao X., Bian H., Liu Y., Zhang M., Zhu X. (2023). Absent in melanoma 2 mediates aging-related cognitive dysfunction by acting on complement-dependent microglial phagocytosis. Aging Cell..

[18] Meng J., Han L., Zheng N., Wang T., Xu H., Jiang Y., Wang Z., Liu Z., Zheng Q., Zhang X. (2022). Microglial Tmem59 Deficiency Impairs Phagocytosis of Synapse and Leads to Autism-Like Behaviors in Mice. J. Neurosci..

[19] Pluvinage J.V., Haney M.S., Smith B.A.H., Sun J., Iram T., Bonanno L., Li L., Lee D.P., Morgens D.W., Yang A.C. (2019). CD22 blockade restores homeostatic microglial phagocytosis in ageing brains. Nature.

[20] Grosso Jasutkar H., Wasserlein E.M., Ishola A., Litt N., Staniszewski A., Arancio O., Yamamoto A. (2024). Adult-onset deactivation of autophagy leads to loss of synapse homeostasis and cognitive impairment, with implications for alzheimer disease. Autophagy.

[21] Gabandé-Rodríguez E., Keane L., Capasso M. (2020). Microglial phagocytosis in aging and Alzheimer’s disease. J. Neurosci. Res..

[22] Sukhorukov V., Voronkov D., Baranich T., Mudzhiri N., Magnaeva A., Illarioshkin S. (2021). Impaired Mitophagy in Neurons and Glial Cells during Aging and Age-Related Disorders. Int. J. Mol. Sci..

[23] Safaiyan S., Kannaiyan N., Snaidero N., Brioschi S., Biber K., Yona S., Edinger A.L., Jung S., Rossner M.J., Simons M. (2016). Age-related myelin degradation burdens the clearance function of microglia during aging. Nat. Neurosci..

[24] Kiani Shabestari S., Morabito S., Danhash E.P., McQuade A., Sanchez J.R., Miyoshi E., Chadarevian J.P., Claes C., Coburn M.A., Hasselmann J. (2022). Absence of microglia promotes diverse pathologies and early lethality in Alzheimer’s disease mice. Cell Rep..

[25] Miao J., Ma H., Yang Y., Liao Y., Lin C., Zheng J., Yu M., Lan J. (2023). Microglia in Alzheimer’s disease: Pathogenesis, mechanisms, and therapeutic potentials. Front. Aging Neurosci..

[26] Dundee J.M., Puigdellívol M., Butler R., Cockram T.O.J., Brown G.C. (2023). P2Y_6_ receptor-dependent microglial phagocytosis of synapses mediates synaptic and memory loss in aging. Aging Cell.

[27] Ni J., Xie Z., Quan Z., Meng J., Qing H. (2024). How brain ’cleaners’ fail: Mechanisms and therapeutic value of microglial phagocytosis in Alzheimer’s disease. Glia.

[28] Liu L., Liu Y., Li N., Huang R., Zheng X., Huang L., Hou S., Yuan Q. (2020). Multiple inflammatory profiles of microglia and altered neuroimages in APP/PS1 transgenic AD mice. Brain Res. Bull..

[29] Behmoaras J., Gil J. (2021). Similarities and interplay between senescent cells and macrophages. J. Cell Biol..

[30] Aman Y., Schmauck-Medina T., Hansen M., Morimoto R.I., Simon A.K., Bjedov I., Palikaras K., Simonsen A., Johansen T., Tavernarakis N. (2021). Autophagy in healthy aging and disease. Nat. Aging.

[31] Lee S.Y., Chung W.S. (2021). The roles of astrocytic phagocytosis in maintaining homeostasis of brains. J. Pharmacol. Sci..

[32] Chung W.S., Verghese P.B., Chakraborty C., Joung J., Hyman B.T., Ulrich J.D., Holtzman D.M., Barres B.A. (2016). Novel allele-dependent role for APOE in controlling the rate of synapse pruning by astrocytes. Proc. Natl. Acad. Sci. USA.

[33] Kono R., Ikegaya Y., Koyama R. (2021). Phagocytic Glial Cells in Brain Homeostasis. Cells.

[34] Rappe A., McWilliams T.G. (2022). Mitophagy in the aging nervous system. Front. Cell Dev. Biol..

[35] Koszła O., Sołek P. (2024). Misfolding and aggregation in neurodegenerative diseases: Protein quality control machinery as potential therapeutic clearance pathways. Cell Commun. Signal..

[36] Jung S., Jeong H., Yu S.W. (2020). Autophagy as a decisive process for cell death. Exp. Mol. Med..

[37] Tabibzadeh S. (2023). Role of autophagy in aging: The good, the bad, and the ugly. Aging Cell..

[38] Liu X., Ye M., Ma L. (2022). The emerging role of autophagy and mitophagy in tauopathies: From pathogenesis to translational implications in Alzheimer’s disease. Front. Aging Neurosci..

[39] Castellazzi M., Patergnani S., Donadio M., Giorgi C., Bonora M., Bosi C., Brombo G., Pugliatti M., Seripa D., Zuliani G. (2019). Autophagy and mitophagy biomarkers are reduced in sera of patients with Alzheimer’s disease and mild cognitive impairment. Sci. Rep..

[40] Collier J.J., Guissart C., Oláhová M., Sasorith S., Piron-Prunier F., Suomi F., Zhang D., Martinez-Lopez N., Leboucq N., Bahr A. (2021). Developmental Consequences of Defective ATG7-Mediated Autophagy in Humans. N. Engl. J. Med..

[41] Nabi S.U., Khan A., Siddiqui E.M., Rehman M.U., Alshahrani S., Arafah A., Mehan S., Alsaffar R.M., Alexiou A., Shen B. (2022). Mechanisms of Mitochondrial Malfunction in Alzheimer’s Disease: New Therapeutic Hope. Oxid. Med. Cell Longev..

[42] Caponio D., Veverová K., Zhang S.Q., Shi L., Wong G., Vyhnalek M., Fang E.F. (2022). Compromised autophagy and mitophagy in brain ageing and Alzheimer’s diseases. Aging Brain..

[43] Zuo H., Chen C., Sa Y. (2023). Therapeutic potential of autophagy in immunity and inflammation: Current and future perspectives. Pharmacol. Rep..

[44] Pradeepkiran J.A., Reddy P.H. (2020). Defective mitophagy in Alzheimer’s disease. Ageing Res. Rev..

[45] Guo J., Huang X., Dou L., Yan M., Shen T., Tang W., Li J. (2022). Aging and aging-related diseases: From molecular mechanisms to interventions and treatments. Signal Transduct. Target. Ther..

[46] Hansen M., Chandra A., Mitic L.L., Onken B., Driscoll M., Kenyon C. (2008). A role for autophagy in the extension of lifespan by dietary restriction in C. elegans. PLoS Genet..

[47] Raz Y., Guerrero-Ros I., Maier A., Slagboom P.E., Atzmon G., Barzilai N., Macian F. (2017). Activation-Induced Autophagy Is Preserved in CD4^+^ T-Cells in Familial Longevity. J. Gerontol. A Biol. Sci. Med. Sci..

[48] Chung K.W., Chung H.Y. (2019). The Effects of Calorie Restriction on Autophagy: Role on Aging Intervention. Nutrients.

[49] Jung S., Choe S., Woo H., Jeong H., An H.K., Moon H., Ryu H.Y., Yeo B.K., Lee Y.W., Choi H. (2020). Autophagic death of neural stem cells mediates chronic stress-induced decline of adult hippocampal neurogenesis and cognitive deficits. Autophagy.

[50] Cao W., Li J., Yang K., Cao D. (2021). An overview of autophagy: Mechanism, regulation and research progress. Bull. Cancer.

[51] Wu H., Che X., Zheng Q., Wu A., Pan K., Shao A., Wu Q., Zhang J., Hong Y. (2014). Caspases: A molecular switch node in the crosstalk between autophagy and apoptosis. Int. J. Biol. Sci..

[52] Kwon Y., Kim J.W., Jeoung J.A., Kim M.S., Kang C. (2017). Autophagy Is Pro-Senescence When Seen in Close-Up, but Anti-Senescence in Long-Shot. Mol. Cells.

[53] Fleming A., Bourdenx M., Fujimaki M., Karabiyik C., Krause G.J., Lopez A., Martín-Segura A., Puri C., Scrivo A., Skidmore J. (2022). The different autophagy degradation pathways and neurodegeneration. Neuron.

[54] Corti O., Blomgren K., Poletti A., Beart P.M. (2020). Autophagy in neurodegeneration: New insights underpinning therapy for neurological diseases. J. Neurochem..

[55] Guo F., Liu X., Cai H., Le W. (2018). Autophagy in neurodegenerative diseases: Pathogenesis and therapy. Brain Pathol..

[56] Zachari M., Longo M., Ganley I.G. (2020). Aberrant autophagosome formation occurs upon small molecule inhibition of ULK1 kinase activity. Life Sci. Alliance..

[57] Gaziev A.I., Abdullaev S., Podlutsky A. (2014). Mitochondrial function and mitochondrial DNA maintenance with advancing age. Biogerontology.

[58] Stavoe A.K.H., Holzbaur E.L.F. (2019). Autophagy in Neurons. Annu. Rev. Cell Dev. Biol..

[59] Liu Y.J., Spangenberg E.E., Tang B., Holmes T.C., Green K.N., Xu X. (2021). Microglia Elimination Increases Neural Circuit Connectivity and Activity in Adult Mouse Cortex. J. Neurosci..

[60] Basilico B., Ferrucci L., Khan A., Di Angelantonio S., Ragozzino D., Reverte I. (2022). What microglia depletion approaches tell us about the role of microglia on synaptic function and behavior. Front. Cell Neurosci..

[61] Bondy S.C., Wu M., Duncan L.T. (2022). Immune function is depressed with aging while inflammation is heightened, a paradox. Advances in Health and Disease, 57.

[62] Greenwood E.K., Brown D.R. (2021). Senescent Microglia: The Key to the Ageing Brain?. Int. J. Mol. Sci..

[63] Norden D.M., Godbout J.P. (2013). Review: Microglia of the aged brain: Primed to be activated and resistant to regulation. Neuropathol. Appl. Neurobiol..

[64] Mirarchi A., Albi E., Beccari T., Arcuri C. (2023). Microglia and Brain Disorders: The Role of Vitamin D and Its Receptor. Int. J. Mol. Sci..

[65] Kou X., Chen D., Chen N. (2020). The Regulation of microRNAs in Alzheimer’s Disease. Front. Neurol..

[66] Chen M.L., Hong C.G., Yue T., Li H.M., Duan R., Hu W.B., Cao J., Wang Z.X., Chen C.Y., Hu X.K. (2021). Inhibition of miR-331-3p and miR-9-5p ameliorates Alzheimer’s disease by enhancing autophagy. Theranostics.

[67] Kou X., Li J., Liu X., Chang J., Zhao Q., Jia S., Fan J., Chen N. (2017). Swimming attenuates d-galactose-induced brain aging via suppressing miR-34a-mediated autophagy impairment and abnormal mitochondrial dynamics. J. Appl. Physiol..

[68] Oudbier S.J., Goh J., Looijaard S.M.L.M., Reijnierse E.M., Meskers C.G.M., Maier A.B. (2022). Pathophysiological Mechanisms Explaining the Association Between Low Skeletal Muscle Mass and Cognitive Function. J. Gerontol. A Biol. Sci. Med. Sci..

[69] Shen K., Liu X., Chen D., Chang J., Zhang Y., Kou X. (2021). Voluntary wheel-running exercise attenuates brain aging of rats through activating miR-130a-mediated autophagy. Brain Res. Bull..

[70] Chen P., Chen F., Lei J., Li Q., Zhou B. (2019). Activation of the miR-34a-Mediated SIRT1/mTOR Signaling Pathway by Urolithin A Attenuates D-Galactose-Induced Brain Aging in Mice. Neurotherapeutics.

[71] Kuerec A.H., Lim X.K., Khoo A.L., Sandalova E., Guan L., Feng L., Maier A.B. (2024). Targeting aging with urolithin A in humans: A systematic review. Ageing Res. Rev..

[72] Zheng J., Hu S., Wang J., Zhang X., Yuan D., Zhang C., Liu C., Wang T., Zhou Z. (2021). Icariin improves brain function decline in aging rats by enhancing neuronal autophagy through the AMPK/mTOR/ULK1 pathway. Pharm. Biol..

[73] Qu W., Li L. (2023). Microglial TREM2 at the Intersection of Brain Aging and Alzheimer’s Disease. Neuroscientist.

[74] Ulland T.K., Song W.M., Huang S.C., Ulrich J.D., Sergushichev A., Beatty W.L., Loboda A.A., Zhou Y., Cairns N.J., Kambal A. (2017). TREM2 Maintains Microglial Metabolic Fitness in Alzheimer’s Disease. Cell.

[75] Moreira O.C., Estébanez B., Martínez-Florez S., de Paz J.A., Cuevas M.J., González-Gallego J. (2017). Mitochondrial Function and Mitophagy in the Elderly: Effects of Exercise. Oxid. Med. Cell Longev..

[76] Jiang X.J., Wu Y.Q., Ma R., Chang Y.M., Li L.L., Zhu J.H., Liu G.P., Li G. (2022). PINK1 Alleviates Cognitive Impairments via Attenuating Pathological Tau Aggregation in a Mouse Model of Tauopathy. Front. Cell Dev. Biol..

[77] Picca A., Faitg J., Auwerx J., Ferrucci L., D’Amico D. (2023). Mitophagy in human health, ageing and disease. Nat. Metab..

[78] Triplett J.C., Tramutola A., Swomley A., Kirk J., Grimes K., Lewis K., Orr M., Rodriguez K., Cai J., Klein J.B. (2015). Age-related changes in the proteostasis network in the brain of the naked mole-rat: Implications promoting healthy longevity. Biochim. Biophys. Acta..

[79] Sadovnikova I.S., Gureev A.P., Ignatyeva D.A., Gryaznova M.V., Chernyshova E.V., Krutskikh E.P., Novikova A.G., Popov V.N. (2021). Nrf2/ARE Activators Improve Memory in Aged Mice via Maintaining of Mitochondrial Quality Control of Brain and the Modulation of Gut Microbiome. Pharmaceuticals.

[80] Singh P., Gollapalli K., Mangiola S., Schranner D., Yusuf M.A., Chamoli M., Shi S.L., Lopes Bastos B., Nair T., Riermeier A. (2023). Taurine deficiency as a driver of aging. Science.

[81] Gruendler R., Hippe B., Sendula Jengic V., Peterlin B., Haslberger A.G. (2020). Nutraceutical Approaches of Autophagy and Neuroinflammation in Alzheimer’s Disease: A Systematic Review. Molecules.

[82] Chen C., Yang C., Wang J., Huang X., Yu H., Li S., Li S., Zhang Z., Liu J., Yang X. (2021). Melatonin ameliorates cognitive deficits through improving mitophagy in a mouse model of Alzheimer’s disease. J. Pineal Res..

[83] Varghese N., Werner S., Grimm A., Eckert A. (2020). Dietary Mitophagy Enhancer: A Strategy Healthy Brain Aging?. Antioxidants.

[84] Makarov M., Korkotian E. (2023). Differential Role of Active Compounds in Mitophagy and Related Neurodegenerative Diseases. Toxins.

[85] Menzies F.M., Fleming A., Caricasole A., Bento C.F., Andrews S.P., Ashkenazi A., Füllgrabe J., Jackson A., Jimenez Sanchez M., Karabiyik C. (2017). Autophagy and Neurodegeneration: Pathogenic Mechanisms and Therapeutic Opportunities. Neuron.

[86] Mishra E., Thakur M.K. (2023). Mitophagy: A promising therapeutic target for neuroprotection during ageing and age-related diseases. Br. J. Pharmacol..

[87] Sala de Oyanguren F.J., Rainey N.E., Moustapha A., Saric A., Sureau F., O’Connor J.E., Petit P.X. (2020). Highlighting Curcumin-Induced Crosstalk between Autophagy and Apoptosis as Supported by Its Specific Subcellular Localization. Cells.

[88] Suárez-Carrillo A., Álvarez-Córdoba M., Romero-González A., Talaverón-Rey M., Povea-Cabello S., Cilleros-Holgado P., Piñero-Pérez R., Reche-López D., Gómez-Fernández D., Romero-Domínguez J.M. (2023). Antioxidants Prevent Iron Accumulation and Lipid Peroxidation, but Do Not Correct Autophagy Dysfunction or Mitochondrial Bioenergetics in Cellular Models of BPAN. Int. J. Mol. Sci..

[89] Chakrabarti L., Eng J., Ivanov N., Garden G.A., La Spada A.R. (2009). Autophagy activation and enhanced mitophagy characterize the Purkinje cells of pcd mice prior to neuronal death. Mol. Brain..

[90] Redza-Dutordoir M., Averill-Bates D.A. (2021). Interactions between reactive oxygen species and autophagy: Special issue: Death mechanisms in cellular homeostasis. Biochim. Biophys. Acta Mol. Cell Res..

[91] Pantelis P., Theocharous G., Lagopati N., Veroutis D., Thanos D.F., Lampoglou G.P., Pippa N., Gatou M.A., Tremi I., Papaspyropoulos A. (2023). The Dual Role of Oxidative-Stress-Induced Autophagy in Cellular Senescence: Comprehension and Therapeutic Approaches. Antioxidants.

[92] Underwood B.R., Imarisio S., Fleming A., Rose C., Krishna G., Heard P., Quick M., Korolchuk V.I., Renna M., Sarkar S. (2010). Antioxidants can inhibit basal autophagy and enhance neurodegeneration in models of polyglutamine disease. Hum. Mol. Genet..

[93] He Y., Wang K., Su N., Yuan C., Zhang N., Hu X., Fu Y., Zhao F. (2024). Microbiota-gut-brain axis in health and neurological disease: Interactions between gut microbiota and the nervous system. J. Cell Mol. Med..

[94] Chen F., Yi W.M., Wang S.Y., Yuan M.H., Wen J., Li H.Y., Zou Q., Liu S., Cai Z.Y. (2022). A long-term high-fat diet influences brain damage and is linked to the activation of HIF-1α/AMPK/mTOR/p70S6K signalling. Front. Neurosci..

[95] Bjedov I., Cochemé H.M., Foley A., Wieser D., Woodling N.S., Castillo-Quan J.I., Norvaisas P., Lujan C., Regan J.C., Toivonen J.M. (2020). Fine-tuning autophagy maximises lifespan and is associated with changes in mitochondrial gene expression in Drosophila. PLoS Genet..

[96] Nixon R.A. (2024). Autophagy-lysosomal-associated neuronal death in neurodegenerative disease. Acta Neuropathol..

[97] Pluta R. (2023). The Dual Role of Autophagy in Postischemic Brain Neurodegeneration of Alzheimer’s Disease Proteinopathy. Int. J. Mol. Sci..

[98] Taban Akça K., Çınar Ayan İ., Çetinkaya S., Miser Salihoğlu E., Süntar İ. (2023). Autophagic mechanisms in longevity intervention: Role of natural active compounds. Expert. Rev. Mol. Med..

